# Determining the severity and prevalence of cybersickness in virtual reality simulations in psychiatry

**DOI:** 10.1186/s41077-025-00358-y

**Published:** 2025-06-04

**Authors:** Amanda Ng, Mai Inagaki, Rachel Antinucci, Sanjeev Sockalingam, Petal S. Abdool

**Affiliations:** 1 Department of Education, Centre for Addiction and Mental Health, Toronto, Canada; 2 Department of Statistical Sciences, University of Toronto, Toronto, Canada; 3 Department of Psychology, University of Toronto Mississauga, Mississauga, Canada; 4 Department of Psychiatry, University of Toronto, Toronto, Canada

**Keywords:** Virtual reality, Cybersickness, Mental health, Psychiatry, Education

## Abstract

**Background:**

The rise in virtual reality (VR) applications in healthcare has introduced immersive VR simulations as a valuable training tool for medical professionals. Despite its advantages, VR use can induce cybersickness, characterized by symptoms such as nausea and disorientation. This study examines the relationship between cybersickness and the degree of physical movement in VR simulations used for psychiatric education.

**Methods:**

The study involved two VR simulations offered at a Canadian mental health hospital: an opioid overdose response (OO) (high movement VR) and suicide risk assessment (SRA) (low movement VR). Participants’ experiences were measured using the Simulator Sickness Questionnaire (SSQ) before and after the training sessions. A nonparametric Mann–Whitney *U*-test was conducted to compare SSQ scores between the two VR simulations.

**Results:**

A total of 91 participants, including healthcare practitioners and students, were involved. The mean SSQ score for the OO training was 4.59/48 (*SD* = 5.78), while for the SRA, it was 3.10/48 (*SD* = 3.48). Mann–Whitney *U*-test revealed a significant increase in nausea scores in OO simulation compared to SRA simulation (*p* = 0.0275), with higher nausea reported in the OO simulation. No significant increases were found in oculomotor symptoms.

**Conclusions:**

Participants in the OO training experienced higher levels of nausea compared to those in the SRA simulation, likely due to increased need for physical movement. These findings underscore the importance of considering the degree of physical movement in the VR training design, specifically the educational value of these movements and the risk of cybersickness negatively impacting VR tolerability for learners.

**Supplementary Information:**

The online version contains supplementary material available at 10.1186/s41077-025-00358-y.

## Background

Virtual reality (VR) applications have seen a rapid increase in popularity over the past few years and are increasingly being incorporated into the mainstream of education and healthcare [1]. Immersive VR refers to the use of a wearable display, such as a head-mounted display (HMD), to present a three-dimensional virtual environment which users navigate using their own position and handheld controllers [2]. One of the greatest benefits of VR is its ability to simulate real-world environments to educate and train professionals. VR simulation allows healthcare professionals to practice their skills and prepare for their roles in an environment that is safe, challenging, and authentic [3].

VR simulations are exceptionally useful for healthcare education and are used to train students and practicing health professionals in areas like surgery, nursing, and more recently mental health [4–8]. Despite its many advantages, there are potential limitations and concerns with the use of VR technology in healthcare education. One limitation is the possibility of VR-induced side effects, specifically cybersickness [9]. Cybersickness is experienced by 20–95% of users, and symptoms range from headache and nausea to stomach discomfort and disorientation [10]. Although the exact reason for cybersickness is still unknown, numerous theories have been proposed to explain this phenomenon. One such theory is the sensory conflict theory, which states that cybersickness is caused by a mismatch between the visual and vestibular systems [11]. This mismatch occurs between the motion that is visually perceived by the user and the lack of corresponding movement in the body. Another theory is the postural instability theory, which states that the loss of postural control during VR immersion may cause cybersickness [12].

Studies have also investigated the relationship of standing or sitting to cybersickness [13–15], the role of head movements [16, 17], visual system [18, 19], and time of exposure to VR [20]. However, there is a dearth of literature studying the impact of physical movement in causing cybersickness. A study by Ciazynska et al. [21] found that increasing the intensity of activity under VR exposure resulted in greater cybersickness. Studies also suggest that VR may be more significant in causing symptoms of nausea over oculomotor disturbance [13, 22], which are the two symptom clusters of the Simulator Sickness Questionnaire (SSQ), a common measure used to assess this phenomenon. Specifically, there is limited research on how cybersickness affects psychiatric education, highlighting the need for studies to explore this gap and its implications for training healthcare professionals.

The following observational study aimed to examine the relationship between cybersickness (nausea and oculomotor symptoms) and degree of physical movement in an immersive VR simulation-based psychiatric education training. We compared experiences and SSQ scores, a measure of simulator sickness symptoms, for participants who had none or slight cybersickness symptoms at baseline, under two different VR simulations that differ in their level of physical movement required to participate. The opioid overdose response training (OO) (high level of physical movement) requires participants to stand and move around, and the suicide risk assessment (SRA) (low level of physical movement) can be done seated or standing, involving limited movement. While the comparison of two distinct VR environments varies multiple parameters, it also provides a comprehensive understanding of VR applications in diverse contexts, highlighting both the practical implications and the inherent challenges in achieving internal validity. In the fast-paced environment of medical education, such observational studies are crucial for quickly advancing our understanding and application of VR technologies.

Based on the published literature [13, 14, 21], we hypothesized that participants with baseline SSQ scores of none or slight in the OO VR training, which requires more physical motion than the SRA VR training, would experience greater cybersickness symptoms (nausea and oculomotor symptoms), especially in the category of nausea.

## Methods

We designed two immersive VR simulation programs at a Canadian mental health hospital: a high physical movement VR OO simulation and a low physical movement VR SRA simulation. Both programs utilized an identical VR headset and two handheld controllers. Both simulation programs were offered as a training resource for healthcare practitioners and students.

### OO description

The OO aimed to teach participants how to effectively administer naloxone, a lifesaving treatment that temporarily reverses the effects of an opioid overdose, in a clinical and community setting. In the community setting, a young, female avatar was found unconscious in her bedroom, and in the clinical setting, a man was found unconscious in the bathroom of a clinic. Participants began the training by assessing the situation and identifying any immediate hazards. Next, participants checked for signs of a potentially fatal overdose by examining areas on the body where signs of an overdose could emerge — specifically the pupils, lips, and fingernails — of the unconscious avatar. Participants then administered naloxone treatment to the unconscious avatars. A naloxone nasal spray was administered in the community setting and an injectable naloxone in the clinical setting. Finally, participants were trained on how to communicate with and provide support to the individual until professional help arrived. The OO simulation required a high level of mobilization from participants, including moving around and squatting down to reach the unconscious individual. Participants were standing for the entire training.

### SRA description

The SRA training provided participants with an opportunity to conduct a suicide risk assessment in a clinic with two different avatar patients. Each scenario included an avatar preceptor who guided participants throughout the VR experience. The participants first learned the core concepts of SRA during a pre-briefing with an avatar preceptor, including the 4Ps framework (predisposing, precipitating, perpetuating, and protective factors) for assessing suicide risk. Participants then interviewed the patient avatar by selecting from a variety of pre-written questions that uncovered risk and protective factors. Finally, the participants categorized each of the uncovered factors using a case formulation framework to determine the suicide risk of each patient. Feedback was continuously provided by the virtual preceptor, real-time pop-ups, and through a variety of verbal and nonverbal responses from the patients. The SRA simulation setting required a moderate level of movement from participants, including turning their bodies, moving their hands or stepping forward to select items. However, participants could also complete the entire training in a stationary position.

### Participant recruitment

Prior to putting on their headsets for the OO and SRA, participants were given a pre-brief to orient them to the VR headset and hardware, to establish psychological safety, and to discuss any possible adverse effects associated with VR. A debrief session was conducted after the VR simulation to consolidate the skills learned in the training to apply them to future clinical interactions. IRB/Quality Project Ethics Review approval was obtained prior to commencing the project. Recruitment occurred from January 2023 to May 2024. Learners were recruited through existing partnerships, the hospital’s student center and advertisements. Participation was voluntary, and detailed at the beginning of the surveys was the purpose, confidentiality statement, data storage strategy, and informed consent statement.

### Simulator Sickness Questionnaire (SSQ)

In the pre-training survey, participants completed the SSQ. The SSQ is a validated tool that measures the severity of motion sickness symptoms experienced by simulator users [23]. It asks participants to rate the level of adverse side effects in terms of 16 symptoms, such as headache, eye strain, nausea, dizziness, and difficulty concentrating [23]. These 16 items are scored on a 4-point scale where 0 = none, 1 = slight, 2 = moderate, and 3 = severe [23]. According to Bouchard et al. [24], a total raw SSQ score of “slight” across all 16 items equates to a subjective cut-off score of 16. Participants who reported “moderate” or “severe” levels on any of the 16 SSQ items in the pre-training survey were excluded from this study to remove individuals who reported adverse side effects before even participating in the VR. Similarly, the post-training survey also included SSQ to measure effects due to VR simulations in this study. Traditionally, the SSQ scores were divided into three subcategories (nausea, oculomotor, and disorientation) and used a weighted scoring system [23]. This often resulted in some items appearing in multiple subcategories [23]. Specifically, the item “nausea” is double counted in nausea and disorientation subcategories, while “difficulty concentrating” and “blurred vision” are double counted in oculomotor and disorientation [23]. To minimize double counting items in each subcategory and ensure equal representation of each type of side effect, this study only used the raw scores from two subcategories (nausea and oculomotor) to score participant SSQ [24]. This approach is further supported by findings from confirmatory factor analysis in recent literature, which indicate that a two-factor model, comprising nausea and oculomotor subcategories, better reflects the structure of symptoms in virtual reality contexts, as opposed to the traditional three-factor model [25]. The nausea category included items 1 (general discomfort), 6 (salivation increasing), 7 (sweating), 8 (nausea), 12 (dizziness with eyes open), 13 (dizziness with eyes closed), 14 (vertigo), 15 (stomach awareness), and 16 (burping). The oculomotor category included items 2 (fatigue), 3 (headache), 4 (eye strain), 5 (difficulty focusing), 9 (difficulty concentrating), 10 (fullness of the head), and 11 (blurred vision).

### Statistical analysis

To assess the impact of movement in a VR simulation on cybersickness, an independent one-sided Mann–Whitney *U*-test was conducted to test if the post-training SSQ scores (nausea scores, oculomotor scores, and total scores) are significantly higher in OO simulation compared to SRA simulation. The Mann–Whitney *U*-test measures if the distributions of both groups are identical without any distributional assumptions. We opted for the one-sided test since OO, which requires a higher level of movement, is likely to result in higher SSQ scores. Therefore, our alternative hypothesis is that OO scores’ distributions are stochastically greater than corresponding SRA scores’ distributions.

## Results

### Participants

Forty-two participants engaged in the OO, and 49 participants engaged in the SRA training. These participants included community work volunteers, psychiatry residents, physicians, and students of nursing and pharmacy.

### Overall results

**Fig. 1:**
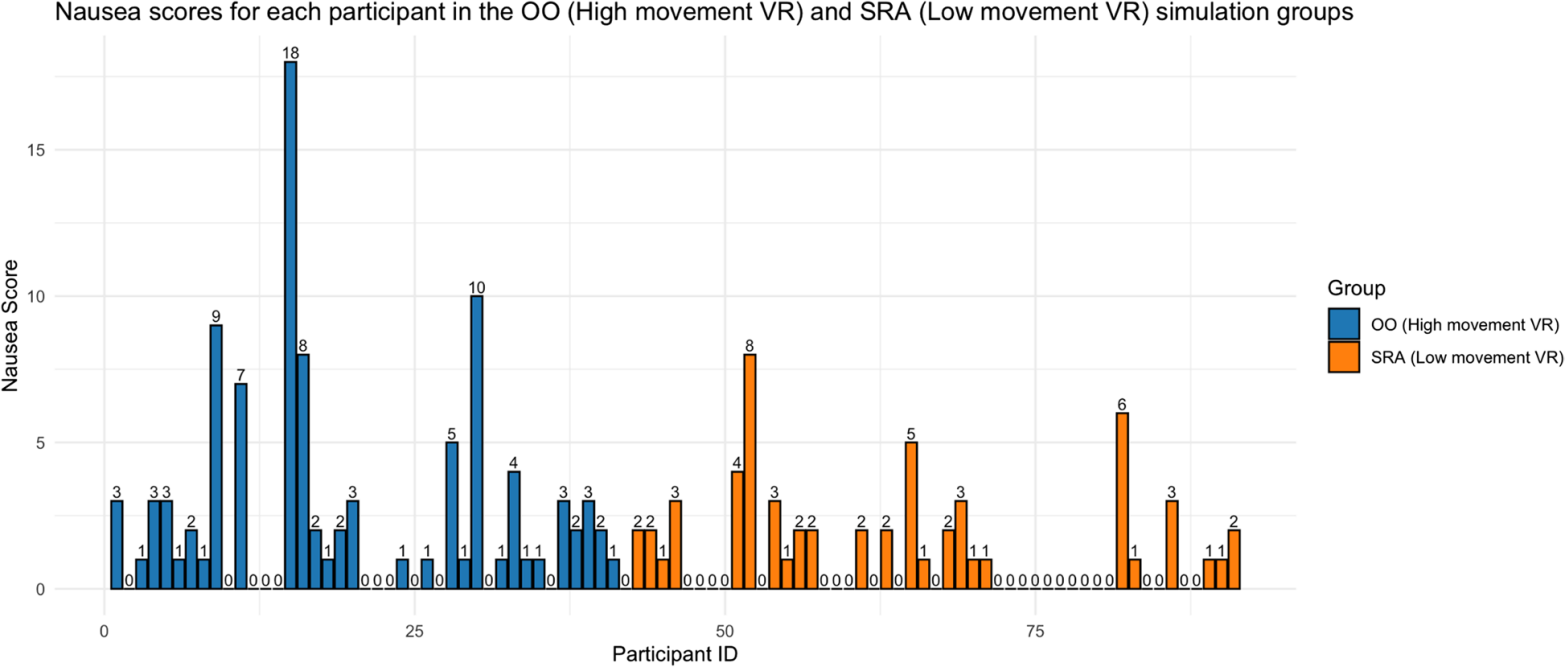
Nausea scores for each participant in the OO (high movement VR) and SRA (low movement VR) simulation groups. Nausea scores were calculated by summing the scores of 9 specific items from the SSQ: 1 (general discomfort), 6 (salivation increasing), 7 (sweating), 8 (nausea), 12 (dizziness with eyes open), 13 (dizziness with eyes closed), 14 (vertigo), 15 (stomach awareness), and 16 (burping). Each bar represents an individual participant, with participant IDs shown along the x-axis and individual nausea scores labeled above each bar

In terms of tolerability, both the OO training and SRA simulations’ overall SSQ scores indicated that most participants experienced little to no simulator sickness symptoms. The mean total SSQ score for VR OO training was 4.59/48 (*SD* = 5.78), with a median score of 3/48. The mean total SSQ score for VR SRA training was 3.10/48 (*SD* = 3.48), with a median of 2/48. See Table 1 for further details. Notably, 9 participants (21.43%) and 15 participants (35.71%) had a score of 0 in the OO training and SRA groups, respectively. In the OO training, 32 participants (76.19%) had a raw SSQ score between 1 and 16, suggesting only “slight” VR side effect symptoms. Additionally, one participant (2.38%) reported a raw score between 17 and 32, indicating “moderate” symptoms. No participants experienced “severe” symptoms. In the SRA simulation, 34 participants (69.39%) indicated “slight” VR side effect symptoms. No participants reported “moderate” or “severe” symptoms. See Fig. 1 for further details.

**Table 1:** Summary statistics of SSQ subcategories and total scores in SRA and OO groups

	Mean	Median	Standard error
SRA (*n* = 49)			
Nausea	1.20	0	1.74
Oculomotor	1.90	1	2.11
Total SSQ	3.10	2	3.48
OO (*n* = 42)			
Nausea	2.38	1	3.51
Oculomotor	2.21	1.5	2.82
Total SSQ	4.59	3	5.78

Table 1 presents the mean, median, and standard error values for the SSQ subcategories (nausea and oculomotor) as well as the total SSQ scores in two participant groups: SRA (*n* = 49) and OO (*n* = 42). The SRA condition, which involves lower physical movement demands, resulted in lower mean scores across all SSQ measures compared to the OO condition.

### Comparison of scores between OO and SRA groups

A comparison of the scores for the nausea side effect subscale was significantly higher for the OO simulation versus the SRA simulation (*OO* 2.38 ± 3.51, *SRA* 1.20 ± 1.74; *p*-value = 0.0275). As for oculomotor side effects subscale scores, there was no significant difference between the OO simulation (*OO* 2.21 ± 2.82) in comparison to the SRA simulation (1.90 ± 2.11) (*p*-value = 0.4069). The difference in the total raw SSQ scores between OO and SRA simulation was not statistically significant (*p*-value = 0.1141). See Table 2 for further details.

**Table 2:** Mann–Whitney *U*-test results and effect sizes

Scores	*U*-statistic	*p*-value	Common language effect size (*f*)
Nausea	1260	0.0275	61.22%
Oculomotor	1058	0.4069	51.41%
Total SSQ	1179	0.1141	57.29%

Table 2 summarizes the results of Mann–Whitney *U*-tests comparing SSQ scores between the SRA and OO groups, along with their corresponding common language effect sizes (*f*). The only statistically significant difference was observed in the nausea subcategory (*U* = 1260, *p* = 0.0275), with a moderate effect size (*f* = 61.22%), indicating that participants in the SRA group reported lower nausea symptoms more frequently than those in the OO group. No significant differences were found for oculomotor symptoms (*p* = 0.4069) or total SSQ scores (*p* = 0.1141).

## Discussion

This study found that participants in the VR OO training experienced significantly higher levels of nausea compared to those training in the VR SRA simulation. A notable difference between these two VR simulation programs is the level of physical movement involved in the training. The OO program involved bending down, shaking the virtual patient, and administering treatment unlike the SRA simulation. These in-game movements might have heightened the sense of virtual-physical motion discrepancy, which could have exacerbated nausea. The nature of movement in VR is hypothesized to be the main reason for cybersickness [26]. Our results build on the current findings by showing a statistically significant connection between increased physical movement and cybersickness.

Additionally, the study found that oculomotor symptoms, such as eye strain, headaches, and blurred vision, were not significantly different between the two VR simulations. In this instance, movement only impacted symptoms of nausea. There have been studies that suggest that VR may induce some SSQ symptoms more than others. Specifically, there seems to be a discrepancy in the literature regarding whether nausea or oculomotor symptoms are a more significant contributor to cybersickness [13, 22, 27]. Our results, though based on an observational approach, demonstrate that in-game movement could differentially impact these SSQ symptoms.

One possible explanation for this finding is that both training programs might impose similar visual demands on participants. Regardless of the specific scenarios presented in each simulation, the requirement to maintain focus on the VR environment, process visual information, and navigate the virtual space may place comparable levels of strain on the visual system. As a result, the oculomotor symptoms might not differ significantly between OO and SRA.

Overall, these findings are important for understanding cybersickness in the context of VR training in mental health and could also be generalized to broader VR simulation design in medical education. This study is the first of its kind that specifically compares the cybersickness of two different VR trainings in psychiatric medical education. By exploring two VR trainings in diverse contexts, a broader understanding of how different factors interact and affect outcomes was analyzed. Understanding which factors exacerbate or trigger cybersickness can inform the future design of VR training programs, boosting their practical utility. Additionally, developing desktop versions of high movement VR trainings can be beneficial for individuals who experience cybersickness. This paper can serve as a guide to those embarking on designing a VR experience.

The use of immersive VR simulation in psychiatric medical education is an innovation that can significantly enrich learner experience through a cyclical process of experiential learning. Kolb’s cycle of experiential learning outlines four stages of learning: concrete experience, reflective observation, abstract conceptualization, and active experimentation [28]. Despite differences in movement levels, both VR SRA and VR OO align with Kolb’s theory and allow learners to progress through all four stages. Active experimentation, in particular, is facilitated by the interactive nature of VR, enabling learners to apply their knowledge in a simulated environment [28]. However, studies have shown that usability of VR can hinder learning outcomes [29, 30]. Considering the impact of movement on VR usability versus the associated educational trade-offs is crucial.

The study had several limitations that are important to acknowledge. First, the comparison of two distinct VR environments reduces internal validity. Second, data from people who had moderate or severe cybersickness symptoms before participating in the VR training were excluded from the study. Excluding this group of learners from this study may have narrowed the generalizability of the findings. This smaller sample size also limits the ability to detect subtle effects and interactions, potentially undermining the overall validity of the conclusions drawn from the study. Individual differences in prior exposure to VR, age, and technological familiarity may have also contributed to variability in cybersickness symptoms. Furthermore, we did not document whether participants were standing or remained seated throughout each VR training. This is a limitation especially since studies have identified that remaining seated or standing could be a potential factor that influences participants’ cybersickness symptoms [13, 15, 31].

## Conclusion

Understanding the relationship between cybersickness and the degree of movement in VR environments can inform decisions on the design and use of VR simulation-based training in psychiatric medical education. This knowledge could be used to determine what educational programs should implement VR simulation training, whether such training methods are necessary to achieve education program objectives, and whether the educational value of high physical movement VR simulation outweighs the risk of potential side effects. For example, a VR simulation training program that incorporates a high degree of in-game motion may require a desktop version to accommodate participants with diverse needs.

Furthermore, knowledge of factors that cause cybersickness is crucial in the design of future VR training to minimize the risk of cybersickness. The insights gained from this study can help guide the identification of the optimal level of movement in VR programs. This would ensure that educational benefits are balanced with user comfort and tolerability, which can not only improve the overall effectiveness of the program but also could increase learner engagement and increase accessibility of VR simulation. To address current limitations, future research should include a range of participants with differential tolerance levels while clearly distinguishing between those who are seated and those who are standing during participation. Expanding the sample size and incorporating diverse learner profiles will enhance the generalizability of findings. Additionally, integrating motion tracking technologies or wearable devices could provide more precise data on physical movement. Future research may also explore strategies to reduce cybersickness, as well as investigating long-term learner engagement and skill retention in VR training for medical education.

## Supplementary Information

Supplementary Material 1. Appendix A.VR Devices and Software Information. Appendix B. VR Opioid Overdose Photos. Appendix C. VR Suicide Risk Assessment Photos. Appendix D. Standardized Instructions VR OO. Appendix E. Standardized Instructions for VR SRA. Appendix F. In-game movements for VR OO. Appendix G. In-game movements for VR SRA.

## Data Availability

The datasets used and/or analyzed during the current study are available from the corresponding author on reasonable request.

## Abbreviations

VR: Virtual reality
HMD: Head-mounted display
SSQ: Simulator Sickness Questionnaire
OO: Opioid overdose
SRA: Suicide risk assessment
