# Supplementary material for: Determining the severity and prevalence of cybersickness in virtual reality simulations in psychiatry
Source: Adv Simul (Lond). 2025 Jun 4;10:32. doi: 10.1186/s41077-025-00358-y (PMC12139111; doi:10.1186/s41077-025-00358-y)
Supplement: Supplementary file 1 — Supplementary Material 1. Appendix A.VR Devices and Software Information. Appendix B. VR Opioid Overdose Photos. Appendix C. VR Suicide Risk Assessment Photos. Appendix D. Standardized Instructions VR OO. Appendix E. Standardized Instructions for VR SRA. Appendix F. In-game movements for VR OO. Appendix G. In-game movements for VR SRA. [file 41077_2025_358_MOESM1_ESM.docx]

**Appendix A-VR Devices and Software Information**

- Meta Quest 2, 2022 Meta Platforms Technologies
- Headset model number: KW49CM
- Controller model number: JD96CX (right), LX39EM (left)
- PN: 891-00296-02
- OS: Android 10
- Builds 58-63 (source [Meta Quest release notes | Quest Help | Meta Store](https://www.meta.com/help/quest/172903867975450/))
- Game version: Final version

**Appendix B- VR Opioid Overdose Photos**

**Clinical Setting:**


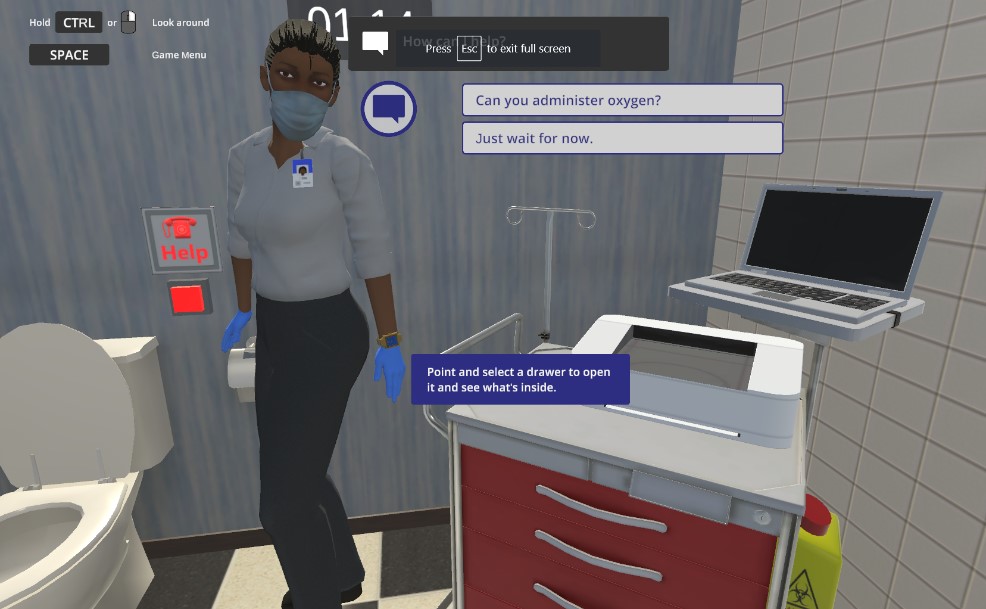


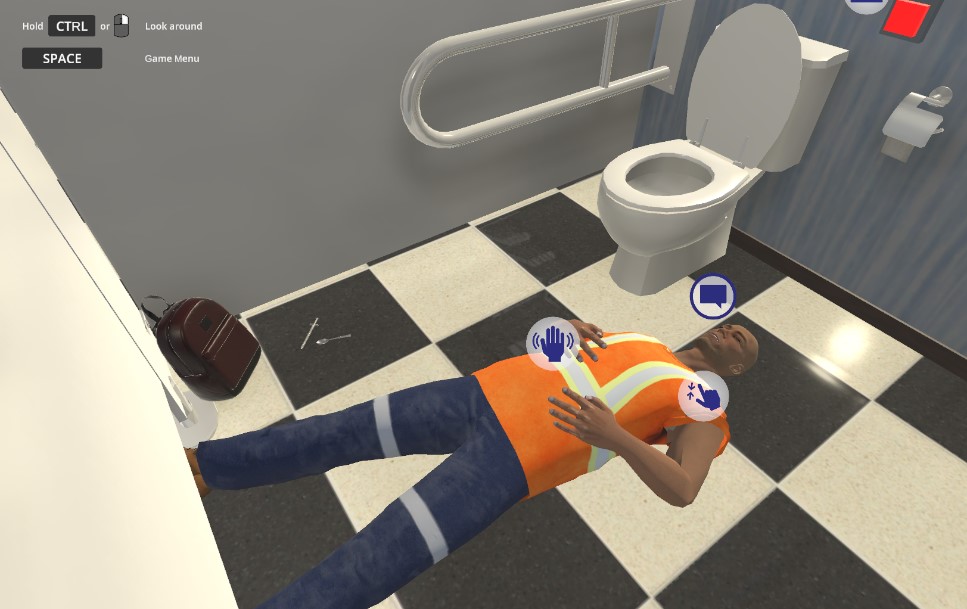


**Community Setting:**


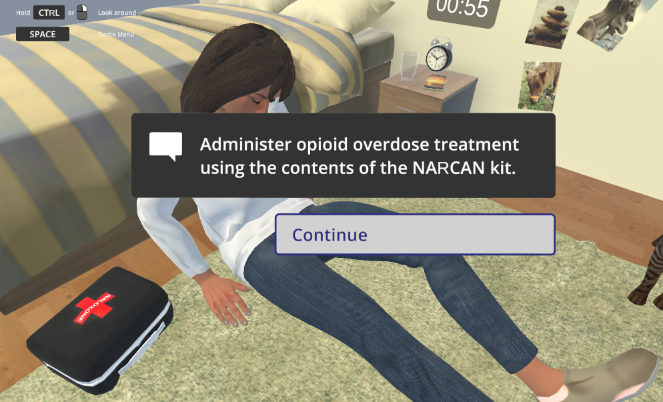


**Appendix C- VR Suicide Risk Assessment Photos**


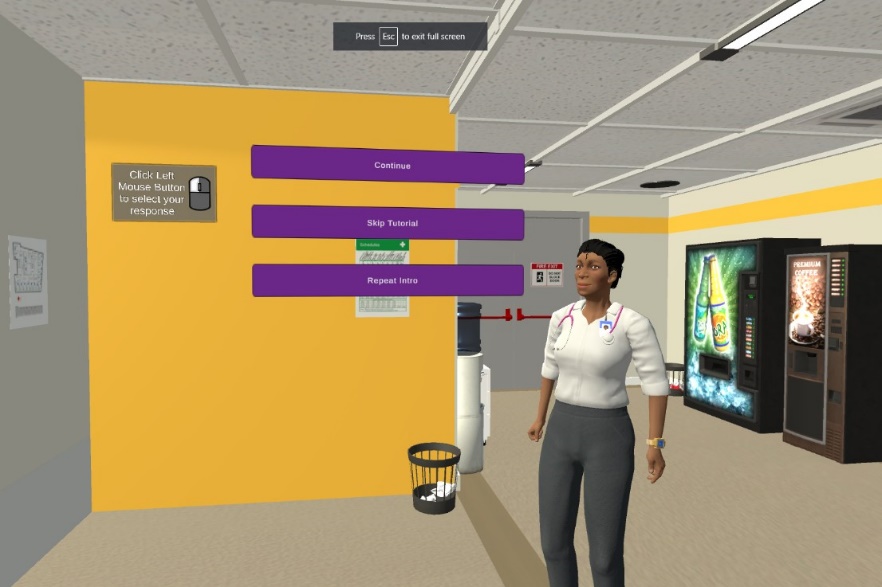


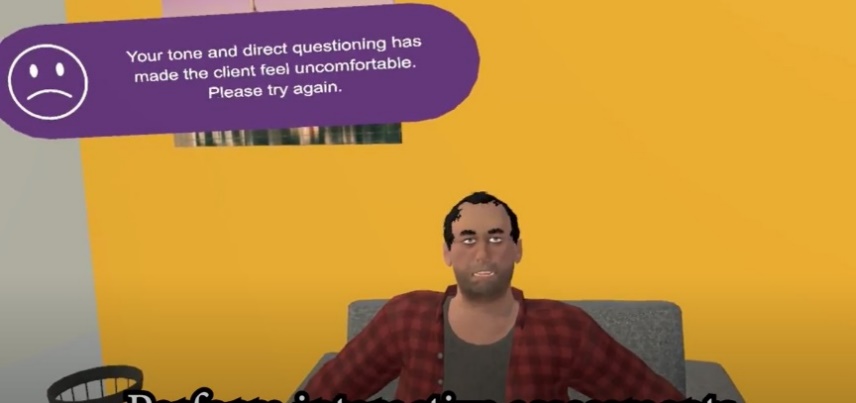

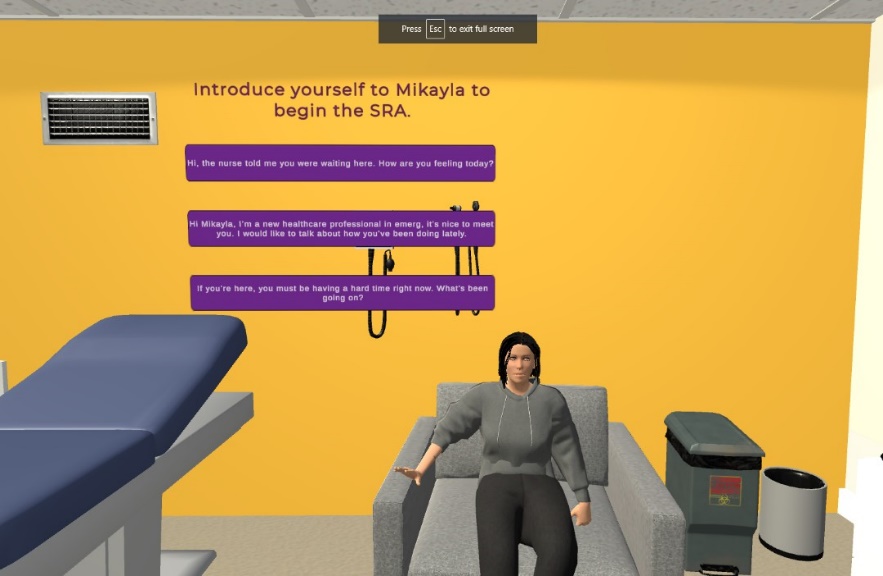


**Appendix D- Standardized Instructions VR OO**

Learners are provided with comprehensive instructions on using the VR OO, including orientation to both the controllers and the headset. They are guided on adjusting the headset straps for a secure and comfortable fit, as well as configuring the eyepiece using its three settings to ensure optimal visual clarity. In terms of the controllers, the following information is provided:

| **#** | **Name** | **Simulation Controls** |
| --- | --- | --- |
| 1 | **Joysticks** | Left joystick – moving body  Right joystick – moving view |
| 2 | **Menu Button** | N/A |
| 3 | **Oculus button** | N/A |
| 4 | **Battery covers** | N/A |
| 5 | **Grip buttons** | Press and hold to grab objects, people. |
| 6 | **Triggers** | Point and click to select dialogue, or options. Point and click to open cart drawers. Combine with #5 on same controller to knock. |


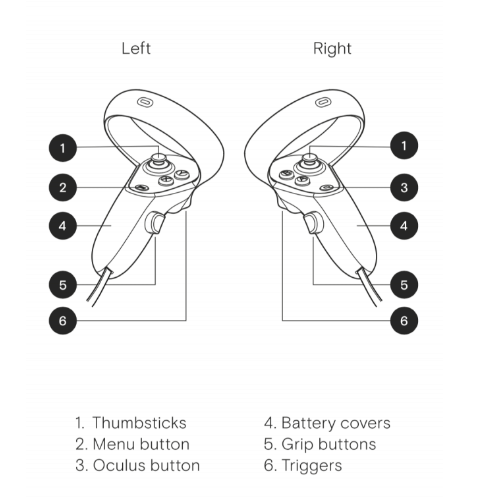


In VR OO, once we have oriented the learners to the headset and controllers (we share the above info from the diagram), we instruct them to complete a Tutorial and then engage either the Clinical or Community Scenario.

**We say:**

- Start by selecting "Tutorial" to practice moving in the virtual environment. When ready, click "Play" and choose either Clinical Scenario or Community Scenario.
- The Clinical Scenario takes place in a healthcare facility bathroom, where a nurse arrives with a crash cart and administers naloxone via syringe.  The Community Scenario takes place in a home shared with a housemate, with no external support during the scenario. Naloxone is administered via nasal spray.
- Then you will pick either "Guided Mode," which provides feedback at set points, or "Unguided Mode," which gives feedback at the end.

**Tutorial for VR OO**

Guides learners in navigating the virtual environment and using the controllers. Tasks include discarding garbage, interacting with doors, using dialogue options, opening drawers, and completing a simulated medical procedure involving syringe preparation and administration to a manikin.

**Scenarios Introductions for VR OO:**

There are two environments to choose from: clinical and community. All scenarios include these stages: communicating with the person, assessing environmental hazards, checking consciousness and calling for help, evaluating symptoms, administering naloxone, and offering support as the person awakens.

**Guided and Unguided Mode Introductions VR OO:**

Each scenario can be played in *Guided* or *Unguided* mode. Guided mode restricts learners' actions to their current stage, preventing further assessments until earlier stages are completed. Unguided mode has no restrictions on the learners.

**Prebrief:**

**The fiction contract:**

In the VR environment, you’ll interact with one of two avatar patients. While the simulation may not mirror clinical practice (e.g. exact phrasing of patient interview question), we ask that you engage with the avatar as if they were real to get the most out of the learning experience.

**Rules of engagement:**

After this prebrief, you’ll complete the simulation at your own pace, followed by a group debrief to reflect on the experience and its application to your practice.

**Ground Rules for learner expectations:**

This virtual simulation offers a safe space to explore, make mistakes, and reflect on your decisions during the debrief.

**Confidentiality:**

We ask that everyone here shares what they learned, but not others’ comments. Evaluation data is anonymous and will be processed by research coordinator.

**Simulation learning objectives:**

Feedback is provided in game based on these stages.

By the end of the VR Opioid Overdose simulation, learners will be able to:​

​

- Assess the situation
- Identify an opioid overdose and call for help
- Administer the required treatment and monitor the patient’s response
- Support the patient after an overdose.

**Sensitive content:**

This simulation is for mature learners who may respond to opioid overdoses and includes emotionally challenging content. Engage bravely and empathetically, take breaks if needed, and seek support from faculty or the resources discussed in the didactic session. Please also reach out to faculty as needed.

**Debrief:**

This debrief will follow the PEARLS framework, (Promoting Excellence and Reflective Learning in Simulation), starting with a description of the scenario and your reactions, followed by an analysis of your experience. We'll also discuss relevant policies and practices and how what you have learned in the simulation fits within this context.

**Appendix E- Standardized Instructions for VR SRA**

**Pre-brief:**

**Scenario Explanation:**

After welcomes and introductions we share:

There are two VR avatar scenarios, Mikayla and Rob that you will work through at your own pace, over the next hour.  You will be conducting suicide risk assessments.

The scenarios begin with a virtual clinical supervisor explaining a Suicide Risk Assessment. You will then be virtually transported into a room where the patient avatar to located.  Once in the virtual room, you will conduct a suicide risk assessment.  Afterward, you’ll review your findings with the virtual clinical supervisor, sort risk factors, and determine risk level, then repeat the process with the second avatar patient, if time allows.

Lastly, there will be a debrief after everyone has engaged in the VR SRA simulation.

**Learning Objectives:**

We say:

1. Demonstrate the process of building a therapeutic alliance with the patient. ​
2. Identify risk and protective factors by conducting a suicide risk assessment.​
3. Organize the factors collected into the 4Ps. ​
4. Appraise which collected factors are potentially modifiable

**Basic Assumption:**

We say:

The assumption is that we possess relevant knowledge, act with positive intent, strive for professional excellence, and are committed to ongoing growth and improvement.

**The fiction contract:**

We say:

We’ve made every effort to create a realistic simulation within a virtual environment that resembles a clinical setting. While it may not be exactly the same as real-life practice, suspending disbelief can make it feel authentic.

We acknowledge that the wording of questions in the Suicide Risk Assessment may differ from your usual practice, and the sequence of available questions is determined by prior selections. We encourage you to engage with the avatar patient as if they were real to maximize your learning experience.

**Active Participation:**

We say:

This is not an assessment—feel free to explore the scenarios as you wish.

**Confidentiality:**

We say:

We ask that everyone here shares what they learned, but not others comments. Evaluation data is anonymous and will be processed by our research coordinator.

**Sensitive content:**

We say:

This is a simulation of suicide risk assessment, so you’ll be asking two virtual patients about their histories and suicidal ideation. It is designed for mature learners who are healthcare providers and includes emotionally challenging content. Engage bravely and empathetically, take breaks if needed, and seek support from faculty or the resources discussed in the didactic session.

**Possible health risks:**

We share & explain:
Potential health risks associated with wearing a VR headset:

- Anxiety
- Nausea
- Eye strain
- ‘VR sickness’
- Possible change in sensory, motor or perceptual abilities
- Epileptic seizures in susceptible people

**Simulation:**

Standardized Instructions on how to use the headset & controllers (same as OO)

We explain how to the begin the session:

You will start by selecting the  “from the beginning,” option on the menu.  You will be greeted by a virtual clinical supervisor who will explain a suicide risk assessment and then you will be virtually transported into a room where the avatar Robert is located. Upon arrival, you will engage with the avatar Robert to conduct a suicide risk assessment.

Alternately, you may choose the “start from specific patient,” option. Choosing this option allows you to pick which patient avatar you would like to engage with first.

**Debrief:**

We say:

This debrief will follow the PEARLS framework, (Promoting Excellence and Reflective Learning in Simulation), starting with a description of the scenario and your reactions, followed by an analysis of your experience. We will finish with a summary and take-away into practice. We ask that you go through this debrief reflecting on your experience, honestly and bravely.

**Appendix F- In-game movements for VR OO**

# Clinical Scenario Interactions

| **Action** | **Movement** |
| --- | --- |
| Moving towards stall where person’s foot is sticking out | Walking, turning head – both can be replaced by using joysticks |
| Calling out | Select dialogue box with raycast |
| Shaking foot | Bending down, grabbing foot – grabbing foot is done by moving hand over foot and holding a button on the controller |
| Opening door | Move hand over door handle, holding button, and pulling arm back |
| Identify hazardous object | Select object box with raycast |
| Shaking body and trapezius pinch | Bending down, grabbing relevant body parts |
| Calling for help | Press hand against button |
| Assessing for signs and symptoms | Bending down, grabbing relevant body parts , game will zoom in further to highlight appearance of eyes and lips when viewing |
| Interact with virtual staff | Select dialogue box with raycast |
| Open/close medical cart drawers | Select drawer box with raycast |
| Getting items from cart | Grabbing items |
| Administering medication | Bending down, placing item (needles/swabs) over relevant body part and in the case of the needle press controller button to depress plunger |
| Post-awakening support | Step back to give space (can be replaced by using joysticks); select dialogue box with raycast |

| **Action** | **Movement** |
| --- | --- |
| Calling out | Select dialogue box with raycast |
| Going upstairs | Move towards stairs, screen transition to upstairs in bedroom – movement can be replaced with joysticks |
| Identify hazardous object | Select object box with raycast |
| Shaking body and trapezius pinch | Bending down, grabbing relevant body parts - grabbing relevant body part is done by moving hand over relevant body part and holding a button on the controller |
| Calling for help | Grab and hold phone with hand; while holding use raycast to select calling |
| Assessing for signs and symptoms | Bending down, grabbing relevant body parts, game will zoom in further to highlight appearance of eyes and lips when viewing |
| Open/close naloxone kit | Select drawer box with raycast |
| Getting items from naloxone | Grabbing items |
| Administering medication | Bending down, placing item (nasal spray/non-rebreather) over relevant body part and in the case of the nasal spray press controller button to depress plunger |
| Post-awakening support | Step back to give space (can be replaced by using joysticks); select dialogue box with raycast |

Community Scenario Interactions

**Appendix G- In-game movements for VR SRA**

| **Action** | **Movement** |
| --- | --- |
| Menu: start from beginning or start from patient | Select dialogue box with raycast |
| Start or skip introduction | Select dialogue box with raycast |
| Virtual clinician appears and talks about SRA and 4Ps | Select dialogue box with raycast |
| Display patient’s chart | -Look down  -Press controller button 5 and 6 together to flip the page |
| Complete SRA with the patient | -Teleported into treatment room  -Select how to interact with the patient (which questions to ask) by selecting dialogue box with raycast |
| Once SRA is completed, choose option to go back to virtual clinician | Select dialogue box with raycast |
| Virtual clinician reviews 4Ps again | Select dialogue box with raycast |
| 4Ps activity | -Must move to board either physically or by joystick  -Grab yellow stickies and put on board using left controller |
| Which risk factors are modifiable | Grab sickies and put in lower board |
| Level of risk | Use controller to select level of risk |
| Debrief with virtual preceptor | N/A |
